# 5-Caffeoylquinic Acid Ameliorates Cognitive Decline and Reduces Aβ Deposition by Modulating Aβ Clearance Pathways in APP/PS2 Transgenic Mice

**DOI:** 10.3390/nu12020494

**Published:** 2020-02-14

**Authors:** Keiko Ishida, Koichi Misawa, Hitomi Nishimura, Tomoya Hirata, Masaki Yamamoto, Noriyasu Ota

**Affiliations:** Biological Science Research, Kao Corporation, 2606 Akabane, Ichikai-machi, Haga-gun, Tochigi 321-3497, Japan

**Keywords:** 5-caffeoylquinic acid, Alzheimer’s disease, amyloid-β clearance, APP/PS2 mice, aquaporin 4, chlorogenic acid, coffee polyphenol, cognitive function, low-density lipoprotein receptor related protein 1

## Abstract

The accumulation of amyloid β (Aβ) in the brain is a major pathological feature of Alzheimer’s disease (AD). In our previous study, we demonstrated that coffee polyphenols (CPP) prevent cognitive dysfunction and Aβ deposition in the brain of an APP/PS2 transgenic mouse AD model. The underlying mechanisms, however, remain to be elucidated. Here, we investigated the effects of the chronic administration of 5-caffeoylquinic acid (5-CQA), the most abundant component of CPP, on cognitive dysfunction in APP/PS2 mice to identify the role of CPP in Aβ elimination. Relative to the untreated controls, the mice fed a 5-CQA-supplemented diet showed significant improvements in their cognitive function assessed by Y-maze and novel object recognition tests. Histochemical analysis revealed that 5-CQA substantially reduced Aβ plaque formation and neuronal loss in the hippocampi. Moreover, 5-CQA upregulated the gene encoding low-density lipoprotein receptor-related protein 1, an Aβ efflux receptor, and normalized the perivascular localization of aquaporin 4, which facilitates Aβ clearance along the paravascular pathway. These results suggest that 5-CQA reduces Aβ deposition in the brain by modulating the Aβ clearance pathways and ameliorating cognitive decline and neuronal loss in APP/PS2 mice. Thus, 5-CQA may be effective in preventing cognitive dysfunction in AD.

## 1. Introduction

In 2018, the number of people living with dementia reached approximately 50 million, which is expected to increase to 152 million by 2050 [1]. The global estimated cost of dementia in 2018 was US$ 1 trillion, and this number is set to increase [1]. The most common cause of dementia is Alzheimer’s disease (AD), which is characterized by memory and cognitive dysfunction. The prevalence of AD is also increasing globally as the elderly population expands [2,3]. AD has no known cure. Thus, current therapeutic options such as specific acetylcholinesterase inhibitors or low-affinity glutamate NMDA receptor antagonists may delay AD progression but can neither improve nor reverse it. Deposition of amyloid β (Aβ) peptides and aggregation of neurofibrillary tangles in the brain are the major pathological features of AD. They play important roles in AD progression by causing synaptic dysfunction and neuronal cell death [4,5]. Aβ accumulation in the brain is attributed to an imbalance between Aβ production and clearance and triggers a pathological cascade [6]. Aβ is generated by β- and γ-secretases that mediate the proteolysis of amyloid precursor protein (APP). Mutations of the genes encoding these enzymes may result in their upregulation and excessive Aβ production. Alterations in these genes are associated with familial early-onset AD [7,8]. In contrast, impaired Aβ clearance is caused mainly by a reduction in Aβ degradation by proteolytic enzymes such as neprilysin and insulin-degrading enzyme (IDE), diminished transport of Aβ across the blood-brain barrier (BBB) via the carrier low-density lipoprotein receptor-related protein 1 (LRP1), and reduced paravascular clearance of Aβ mediated by cerebrospinal fluid (CSF) transport [9]. Recent studies demonstrated that impaired Aβ clearance rather than excessive Aβ production is a major event in late-onset AD [10,11]. Thus, enhancing Aβ clearance may be an efficacious therapeutic strategy for AD. The failure of recent clinical trials focusing on the inhibition of Aβ accumulation via the aforementioned mechanisms has diverted research attention towards prophylaxis and treatment during the early stages of the disease. The use of nutritional substances such as polyphenols is a potential approach towards early-stage AD therapy.

Coffee is one of the most popular and widely consumed beverages worldwide. Epidemiological studies have indicated that regular coffee drinking may reduce the risk of cognitive disorders such as AD [12,13]. A CAIDE (Cardiovascular Risk Factors, Aging and Dementia) study revealed that drinking three to five cups of coffee daily at midlife was associated with reduced risks of dementia and AD later in life [14]. Coffee contains caffeine, phenolic compounds (coffee polyphenols (CPP)), and other bioactive ingredients. Caffeine may partially account for the beneficial effects of coffee as it is a nonselective adenosine receptor blocker [15]. Several studies disclosed that caffeine intake decreased brain Aβ levels in AD model mice [16,17]. On the other hand, caffeine consumption during pregnancy accelerated cognitive deficits in the offspring of a mouse tauopathy model. Therefore, early-life caffeine exposure may constitute a risk factor for early-onset AD [18]. Caffeine may have both beneficial and detrimental effects on cognitive function. However, coffee also contains abundant phenolic compounds such as chlorogenic acids (CGAs). Seventy to 350 mg of CGAs may be contained in a single 200 mL cup of coffee [19]. The CGAs in coffee comprise caffeoylquinic acids (3-CQA, 4-CQA, and 5-CQA), feruloylquinic acids (3-FQA, 4-FQA, and 5-FQA), and diCQAs (3,4-diCQA, 3,5-diCQA, and 4,5-diCQA). In general, 5-CQA is the most abundant of these in coffee [20,21]. Lower CGA levels have been measured in apples, pears, eggplants, and potatoes. Several reports demonstrated the beneficial effects of CGAs on the central nervous system (CNS). In vitro studies revealed that CGAs promoted neurite outgrowth in rat hippocampus neuronal cells [22] and reduced Aβ-induced neuronal cell death by disaggregation of Aβ fibrils [23,24]. CGAs also presented with inhibitory activity against acetylcholinesterase (AChE) and butyrylcholinesterase (BChE) in rat brain homogenate [25]. A pre-clinical Morris water maze in SAMP8 mice showed that one month of CGA administration improved spatial learning and memory [26]. The antioxidant property of CGAs improved temporary amnesia in mice with scopolamine-induced learning and memory impairment [27]. All of the aforementioned studies tested caffeine-free CGAs. The effects of CGAs on human cognition remain controversial as most clinical studies on them demonstrated the efficacy of coffee as a whole and not that of CGAs alone. However, it was shown that four to six months of CGA intake improved cognitive function in healthy elderly patients [28,29].

In our previous study, we demonstrated that CPP comprising caffeoylquinic acids, feruloylquinic acids, and diCQAs prevented cognitive dysfunction and Aβ deposition in the hippocampi of APP/PS2 mice [30]. APP/PS2 double-transgenic mice are AD models that highly express mutant forms of human Aβ precursor protein (APP) and human presenilin-2 (PS2) [31,32]. Thus, the phenolic compounds in coffee can help prevent the cognitive decline associated with AD. Nevertheless, their underlying mechanisms remain to be elucidated. Here, we show that 5-CQA improved cognitive impairment, Aβ deposition, and neuronal loss in APP/PS2 mice by modulating their Aβ clearance pathways.

## 2. Materials and Methods

### 2.1. Animals and Diets

APP/PS2 double-transgenic mice and their wild type (WT) littermates were produced as described by Toda et al. [32]. Briefly, APPswe mice that express the mutant human APP gene were obtained from Taconic Biosciences (#1349; Hudson, NY, USA). PS2M1 mice that express the mutant human PS2 gene were purchased from Oriental Yeast Co. Ltd. (Tokyo, Japan). Male APP/PS2 mice and female PS2M1 mice were crossed via in vitro fertilization and embryo transfer to produce APP/PS2 double-transgenic mice. Polymerase chain reaction (PCR) analysis of tail tip DNA was used to validate the mouse genotypes.

**Figure 1:**
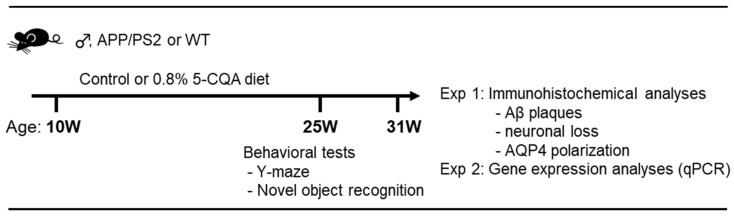
Experimental design. Ten-week-old male APP/PS2 mice and their WT littermates were maintained on control or 0.8% 5-CQA diets. Behavioral tests were performed after four months on these diets. Immunohistochemical analyses and gene expression analyses carried out after six months on these diets.

Male APP/PS2 mice and their WT littermates at 10 weeks of age were maintained at 23 ± 2 °C and 55 ± 10% relative humidity under a 12-h light cycle (0700–1900 h). The mice were assigned to three different groups (*n* = 23−25). One group was used for behavioral studies and immunohistochemistry (IHC) (*n* = 16−18). Another was used for RNA extraction (*n* = 7). The mice had ad libitum water access and were fed either a control or a 5-CQA (#70930; Cayman Chemical, Ann Arbor, MI, USA) diet. The control diet was comprised of 10% (*w*/*w*) fat, 61.5% (*w*/*w*) potato starch, 20% (*w*/*w*) casein, 4% (*w*/*w*) cellulose, 3.5% (*w*/*w*) vitamins, and 1% (*w*/*w*) minerals. The energy content of the control diet was 4.16 kcal g^−1^. The 5-CQA diet comprised the control diet plus 0.8% (*w*/*w*) 5-CQA which included the same polyphenolic quantities of caffeoylquinic acids and di-CQAs as those in the 1% CPP diet used in our prior study [30]. The potato starch content was reduced to 60.7% (*w*/*w*) to compensate for the addition of 5-CQA. The energy content of the 5-CQA diet was 4.13 kcal g^−1^. The mice were maintained on these diets for 21–23 wk (6 mo). Food intake was recorded every 3–4 d, and body weight was measured weekly. The behavioral analysis was conducted after 15–17 wk (4 mo). The experimental design was shown in Figure 1. The protocol (No. S17053-0000) including all animal experiments was approved by the Kao Corporation Animal Care Committee.

### 2.2. Behavioral Analysis

The Y-maze test and then the novel object recognition test were performed after 15–17 wk (four months).

#### 2.2.1. Y-Maze Test

The Y- maze apparatus had three arms separated at 120° angles. Each arm was 38 cm long, 4 cm wide, and 12.5 cm high. The mice were placed in the center of a Y- maze and permitted to explore the maze for 8 min. The number of entries into each arm was visually counted. Alteration was defined as three consecutive entries to each arm. The percentage alteration was calculated as follows:

{[alteration/(total number of arm entries − 2)] × 100}(1)

#### 2.2.2. Novel Object Recognition Test

The novel object recognition test was carried out as previously described [30]. Briefly, for the habituation trial, the mice were permitted to explore an empty opaque plastic box (30 × 30 × 30 cm) for 10 min. For the next-day training trial, each mouse was allowed to explore the test box with two blocks (familiar) for 5 min. One of the blocks was replaced with a new object (novel). Two hours after the training trial, a test trial was conducted for 5 min. The exploration rate for each familiar or novel object during the test trial was calculated as follows:

{[familiar object or novel object/(familiar object + novel object)] × 100}(2)

The discrimination index was presented as follows:

{(novel object - familiar object)/(familiar object + novel object)}(3)

### 2.3. Immunohistochemical Analyses

The brain fixation for immunohistochemical analysis was performed as previously described [30]. Briefly, after 21–23 wk (6 months), the mice brains were transcardially perfused with Dulbecco’s phosphate-buffered saline (DPBS) plus heparin (5 U mL^−1^; Mochida Pharmaceutical, Tokyo, Japan) and 4% (*v*/*v*) paraformaldehyde (PFA) (Wako Pure Chemical Industries, Ltd., Osaka, Japan) under isoflurane anesthesia (Forane^®^; Abbott, Tokyo, Japan). After perfusion, the brains were excised and fixed in 4% (*v*/*v*) PFA at 4 °C. Brain samples were embedded in paraffin and sliced into sections 3-5-µm thick.

#### 2.3.1. Aβ Plaques

Aβ plaque detection was conducted as previously described [30]. In brief, deparaffinized brain tissue sections were incubated with 0.1% (*v*/*v*) hydrogen peroxide to prevent endogenous peroxidation. Monoclonal anti-human Aβ (#10323; 1:200; Immuno-Biological Laboratories, Gunma, Japan) and horseradish peroxidase (HRP)-conjugated streptavidin (Nichirei Biosciences, Tokyo, Japan) were used to detect Aβ plaques. For color development, diaminobenzidine substrate (DAKO, Tokyo, Japan) was used. Acquisition of bright-field images was performed using a microscope (BZ-X710; KEYENCE, Osaka, Japan). BZ-II software (KEYENCE, Osaka, Japan) was used for quantitative image analysis. The average values for the selected regions were quantified using six slices per mouse. The Aβ deposition levels in three areas (97,200 µm^2^; 360 × 270 µm/each area) selected from each cerebral cortex (parietal and primary somatosensory cortex) and hippocampus were computed as the number and percentage area of brain regions presenting with Aβ immunoreactivity.

#### 2.3.2. Nissl Staining

Brain tissue sections were stained with cresyl violet solution for 90 min and scanned under a microscope (BZ-X710; KEYENCE, Osaka, Japan). The average values for the selected regions were quantified using three slices per mouse. The average numbers of Nissl-positive cells were determined for three randomly selected areas (97,200 µm^2^; 360 × 270 µm/each area) in the CA1, CA2, and CA3 regions of the hippocampus. The cells were counted with BZ-II (KEYENCE, Osaka, Japan).

#### 2.3.3. Perivascular Aquaporin 4 (AQP4) Polarization

Endogenous peroxidation was blocked with proteinase K (DAKO, Tokyo, Japan) and 0.1% (*v*/*v*) hydrogen peroxide. Polyclonal anti-rabbit AQP4 (#AB3594; 1:500; Millipore, Temecula, CA, USA) and streptavidin-biotin-labeled secondary antibody (DAKO, Tokyo, Japan) were used for AQP4 detection. For color development, an avidin-biotin complex staining kit (Thermo Fisher Scientific, Waltham, MA, USA) and diaminobenzidine substrate (DAKO, Tokyo, Japan) were used according to the manufacturer’s instructions. The stained sections were counterstained with hematoxylin and visualized under a microscope (BZ-X710; KEYENCE, Osaka, Japan). The images were quantitatively analyzed with BZ-II (KEYENCE, Osaka, Japan). The average values of the selected regions were quantified using six slices per mouse. The AQP4 expression levels in three randomly selected hippocampal areas (97,200 µm^2^; 360 × 270 µm/each area) were computed as the percentage of brain regions presenting with AQP4 immunoreactivity. AQP4 polarization around vessels was determined by calculating the ratios of AQP4 expression in the perivascular and parenchymal domains [33].

### 2.4. RNA Extraction and Quantitative PCR (qPCR)

The cerebral cortices and hippocampi were dissected after transcardial perfusion with DPBS plus heparin as described above. The tissues were frozen and stored at −80 °C until analysis. Total RNA was extracted with the RNeasy Plus Universal Mini Kit (Qiagen, Hilden, Germany) according to the manufacturer’s instructions. A High-Capacity RNA-to-cDNA Kit (Applied Biosystems, Foster City, CA, USA) was used to synthesize cDNAs for real-time qPCR. The qPCR assays were conducted with an Applied Biosystems ViiA7 Real-Time PCR System (Applied Biosystems, Foster City, CA, USA). Pre-designed PCR primers and FAM-labeled TaqMan probes were purchased from Applied Biosystems (TaqMan Gene Expression Assays; Applied Biosystems, Foster City, CA, USA). The glyceraldehyde-3-phosphate dehydrogenase (*GAPDH*) housekeeping gene was used to normalize the expression level of each gene. The genes and TaqMan probes used in this study are listed in Supplementary Table S1.

### 2.5. Statistical Analysis

Data are presented as means ± SEM. Except for the novel object recognition test, all data were analyzed by one-way ANOVA followed by Bonferroni’s post-hoc test or Student’s *t*-test in GraphPad Prism v. 6 (GraphPad Software, La Jolla, CA, USA). For the novel object recognition test, a one-sample *t*-test was used to compare the behavioral scores at the 50% probability level. Differences were considered significant at *p* < 0.05.

## 3. Results

### 3.1. Body Weight and Food Intake

The experimental design was shown in Figure 1. The 5-CQA treatment did not affect the general health of APP/PS2 mice. Table 1 shows body weight and cumulative food intake at four months when the behavioral tests were conducted. Cumulative food intake was lower in the WT group than the APP/PS2 group (Table 1; F_2,69_ = 0.68, *p* < 0.001). Compared to the WT group, APP/PS2 + 5-CQA group showed lower body weight and higher cumulative food intake, but treatment with 5-CQA did not alter body weight or food intake in the APP/PS2 group (n.s.).

### 3.2. Cognitive Function

#### 3.2.1. Y-Maze Test

**Figure 2:**
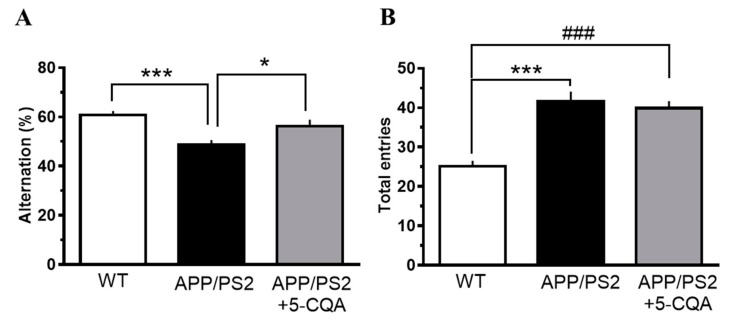
Spatial recognition memory in the Y-maze test. (**A**) percentage alteration. (**B**) total number of maze entries. Mice received experimental diets for 16–17 wk prior to measurements. Data are means ± SEM (*n* = 16–18 mice/treatment). *: *p* < 0.05, ***: *p* < 0.001 vs. APP/PS2, ###: *p* < 0.001 vs. WT (Bonferroni’s post-hoc test).

The effects of 5-CQA on spatial recognition memory were evaluated with a Y-maze test. The percentage alteration was significantly less in the APP/PS2 group (F_2,48_ = 2.74, *p* < 0.001) than that for the WT group (Figure 2A). In contrast, the percentage alteration was significantly greater in the APP/PS2 mice fed the 5-CQA diet (F_2,48_ = 2.74, *p* < 0.05) than that for the APP/PS2 group (Figure 2A). The total number of maze entries during the test had increased in the APP/PS2 mice (F_2,48_ = 1.58, *p* < 0.01) relative to that for the WT mice (Figure 2B). The total number of maze entries for the APP/PS2 + 5-CQA group was not different from that of the APP/PS2 group (n.s.) (Figure 2B).

#### 3.2.2. Novel Object Recognition Test

**Figure 3:**
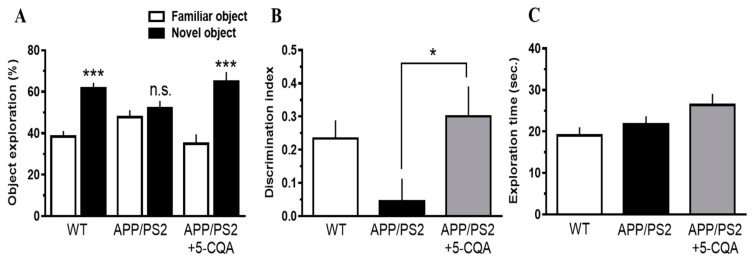
Visual recognition memory assessed by the novel object recognition (NOR) test. (**A**) Exploration rate for each object. (**B**) Discrimination index. (**C**) Total time of the object exploration. Experimental diets were administrated for 16-21 wk prior to measurements. Data are means ±SEM (*n* = 16–18 mice/treatment). (**A**) Statistical significance (***: *p* < 0.005) was assessed by a one-sample *t*-test. (**B**,**C**) Statistical significance was assessed by Bonferroni’s post-hoc test. *: *p* < 0.05 vs. APP/PS2.

The novel object recognition test was carried out to investigate the effects of 5-CQA on visual recognition memory. The exploration times for the two objects were comparable among all three groups in the training trial (data not shown). In the test trial, the percentage novel object exploration time for the WT and APP/PS2 + 5-CQA groups had significantly increased (*p* < 0.005, One-sample *t*-test) compared to that for the familiar object. In contrast, the percentage exploration times for the familiar and novel objects were comparable for the APP/PS2 groups (Figure 3A). The ability to discriminate familiar and novel objects (quantified as the discrimination index) for the APP/PS2 + 5-CQA group had significantly increased (F_2,48_ = 0.82, *p* < 0.05) compared with that for the APP/PS2 group (Figure 3B). The total test trial exploration time did not differ among all three groups (n.s.) (Figure 3C).

### 3.3. Aβ Deposition in the Brain

**Figure 4:**
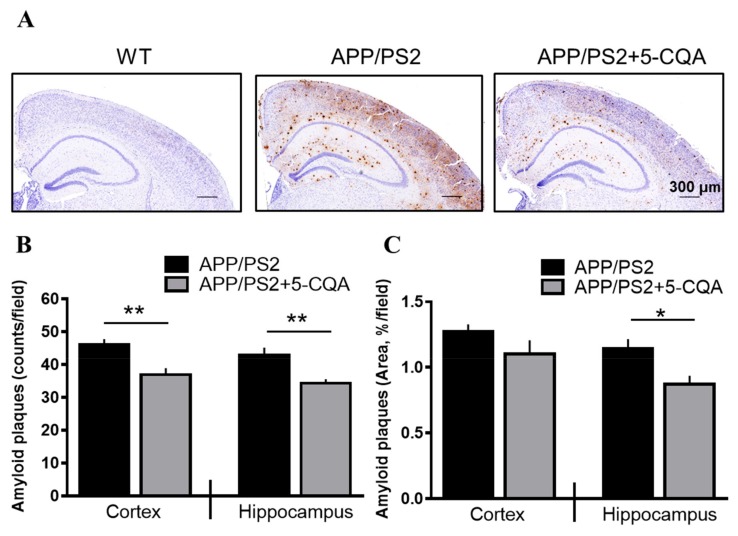
Aβ plaques in APP/PS2 mice brain tissue. (**A**) Aβ-immunoreactivities in WT, APP/PS2, and APP/PS2 + 5-CQA mice. Scale bar: 300 µm at ×40 magnification. (**B**) Number of Aβ plaques in cortex (parietal and primary somatosensory cortex) or hippocampus. (**C**) Area of Aβ plaques in cortex (parietal and primary somatosensory cortex) or hippocampus. Data are means ± SEM (*n* = 6–7 mice/treatment). *: *p* < 0.05, **: *p* < 0.01 vs. APP/PS2 (Student’s *t*-test).

As shown in Figure 4, Aβ plaques were detected in the APP/PS2 mice but not in the WT mice. Significant Aβ deposition was observed in the cortex and hippocampus of the APP/PS2 group. Nevertheless, Aβ deposition was apparently reduced in the APP/PS2 + 5-CQA group (Figure 4A). The numbers of Aβ plaques in the cortex and hippocampus were markedly decreased (*p* < 0.01; Student’s *t*-test) for the APP/PS2 + 5-CQA group relative to those for the APP/PS2 group (Figure 4B). The percentage Aβ plaque area was remarkably reduced (*p* < 0.05; Student’s *t*-test) in the hippocampus of the APP/PS2 + 5-CQA group compared to that for the APP/PS2 group (Figure 4C).

### 3.4. Neuronal Loss in Hippocampus

**Figure 5:**
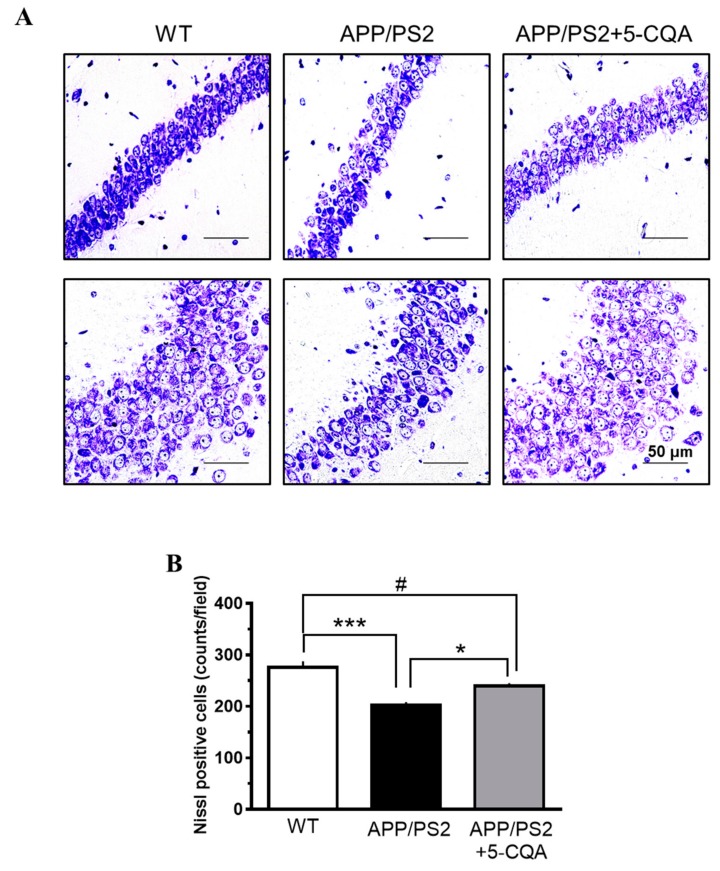
Effects of 5-CQA on neuron loss in APP/PS2 mice hippocampus. (**A**) Nissl-staining neurons in hippocampal CA1, CA2, and CA3 regions of WT, APP/PS2, and APP/PS2 + 5-CQA mice. Scale bar: 50 µm at ×40 magnification. (**B**) Quantification of Nissl-staining neurons. Data are means ± SEM (*n* = 5 mice/treatment). *: *p* < 0.05, ***: *p* < 0.001 vs. APP/PS2, #: *p* < 0.05 vs. WT (Bonferroni’s post-hoc test).

Figure 5 shows Nissl staining of neuropathological changes in the hippocampal CA1, CA2, and CA3 regions. Neuron loss and nuclear disappearance/shrinkage were observed in the APP/PS2 group relative to the WT group. In contrast, neuron loss and nuclear disappearance/shrinkage were relatively decreased in the APP/PS2 + 5-CQA group (Figure 5A). The number of Nissl-positive cells was significantly decreased in the APP/PS2 mice (F_2,12_ = 0.69, *p* < 0.05) compared to that for the WT mice (Figure 5B). In contrast, the number of Nissl-positive cells was significantly increased in the APP/PS2 + 5-CQA group (F_2,12_ = 0.69, *p* < 0.01) relative to that of the APP/PS2 group (Figure 5B).

### 3.5. Aβ Production, Degradation, and Transport-Related Gene Expression

The expression levels of the *APP* genes were comparable between the APP/PS2 and APP/PS2 + 5-CQA groups. The expression levels of *ADAM10* (α-secretase) were similar among all three groups. The expression levels of *BACE1* (β-secretase) were higher in the hippocampus (F_2,18_ =1.57, *p* < 0.01) but not the cortex of the APP/PS2 + 5-CQA group compared to that of the WT group. Treatment with 5-CQA did not alter the expression levels of *BACE1* in the APP/PS2 group. The expression levels of Aβ-degrading genes such as *IDE* and *NEP* were comparable for all three groups. The APP/PS2 + 5-CQA group showed significantly higher mRNA expression of *LRP-1* in the hippocampus but not the cortex compared with the APP/PS2 and WT group (F_2,18_ = 3.15, *p* < 0.05 vs. APP/PS2, *p* < 0.01 vs. WT). The mRNA expression of *RAGE* was higher in the cortex but not the hippocampus of the WT group relative to that of the APP/PS2 group (F_2,18_ = 0.29, *p* < 0.05). The expression levels of *RAGE* in the 5-CQA-treated APP/PS2 and untreated APP/PS2 did not differ (Table 2).

### 3.6. Perivascular AQP4 Polarization

**Figure 6:**
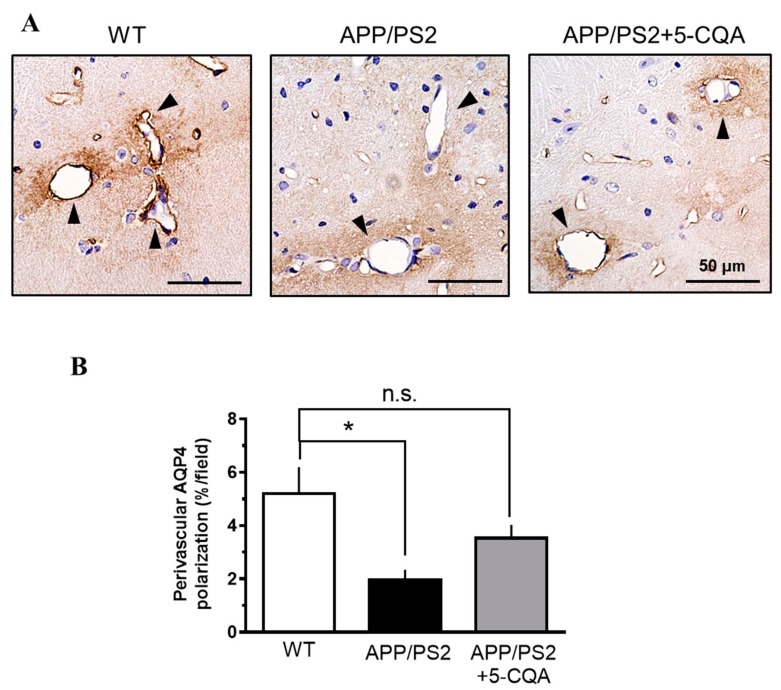
Perivascular AQP4 polarization in APP/PS2 mouse hippocampus. (**A**) Representative AQP4 immunoreactive images of hippocampus in WT, APP/PS2, and APP/PS2 + 5-CQA mice. AQP4 expression closely abutting vessels (black arrows). Scale bar: 50 µm at ×40 magnification. (**B**) Quantification of perivascular AQP4 polarization. Data are means ± SEM (*n* = 6–8 mice/treatment). *: *p* < 0.05 vs. APP/PS2 (Bonferroni’s post-hoc test).

As shown in Figure 6, AQP4 was highly expressed in the regions abutting the vessels (Figure 6A). The percentage of AQP4 polarization around the vessels had significantly diminished in the APP/PS2 group (F_2,18_ = 2.37, *p* < 0.05) compared to that for the WT group (Figure 6B). On the other hand, the percentage of AQP4 polarization around the vessels had been comparable between the WT and the APP/PS2 + 5-CQA group (Figure 6B).

## 4. Discussion

Coffee contains numerous polyphenols such as CGAs, which may have various health benefits including the prevention or mitigation of neurodegenerative diseases. In our previous study, we showed that CPP prevented cognitive decline and Aβ accumulation in APP/PS2 mice [30]. The underlying mechanisms, however, were unclear. In the present study, we demonstrated that chronic ingestion of 5-CQA, the most abundant component of CPP, ameliorated cognitive deficits and prevented Aβ deposition and neuronal loss in APP/PS2 mice. These results are consistent with our earlier research on CPP administration. Thus, 5-CQA is the principle neuroprotective constituent of CPP. To the best of our knowledge, this is the first report to demonstrate the efficacy of 5-CQA on an AD mouse model. We also showed that 5-CQA upregulates *LRP1* expression and normalizes perivascular AQP4 localization in the hippocampus. Therefore, 5-CQA may decrease Aβ accumulation in the brain by modulating the Aβ clearance routes.

Chronic 5-CQA ingestion improved memory loss according to the results of the Y-maze and novel object recognition tests. The Y-maze test is a spatial learning task based on working memory [34], whereas the novel object recognition test evaluates the spatial and temporal context of object recognition and visual recognition memory [34,35]. The hippocampus exhibits initial vulnerability in AD pathology and plays a pivotal role in the formation of these memories [36,37]. It is possible that 5-CQA protects the hippocampus against neuronal dysfunction evoked by Aβ toxicity. Our histochemical analysis disclosed that dietary 5-CQA significantly reduced neuronal loss and Aβ plaques in the hippocampus relative to the untreated. Aβ accumulation and toxicity cause synaptic dysfunction, which leads to neuronal and memory loss in AD pathology [6]. Therefore, a reduction in Aβ plaques in the brain affected by 5-CQA could prevent neuronal loss and cognitive decline.

Potential mechanisms for the decrease in brain Aβ accumulation mediated by 5-CQA administration include the inhibition of excess Aβ production, promotion of Aβ degradation, and enhancement of Aβ clearance. To clarify the modes of action of 5-CQA, we evaluated the expression levels of the mRNAs associated with APP processing and Aβ degradation and transport. The present study showed that 5-CQA upregulated the Aβ efflux receptor *LRP1* but had no influence on the expression levels of APP-processing or Aβ-degrading enzymes. Thus, 5-CQA facilitated Aβ clearance. It was reported that LRP1 participates in both Aβ endocytosis and transcytosis across the BBB [38,39,40]. Recent studies indicated that BBB-mediated clearance is a major route for Aβ elimination in the brain and that LRP1 may be a key receptor for Aβ clearance [39]. Clinical studies on AD patients revealed that they had low brain LRP1 levels [39,41]. In rodents, LRP1 expression in the brain and brain capillaries decreased in an age-dependent manner [42]. A recent study disclosed that relative to the control, brain endothelial cell-specific *LRP1* deletion in mice significantly decreased Aβ efflux, elevated Aβ accumulation in the brain, and exacerbated memory deficits [40]. As LRP1 expression plays a crucial role in Aβ clearance and cognitive function maintenance, *LRP1* upregulation by 5-CQA should effectively eliminate Aβ in the brain and improve cognitive function. In our preliminary data, mRNA expression of *LRP1* was correlated with those related to neuronal function such as Activity-regulated cytoskeleton-associated protein (*Arc*) and *synaptophysin* in APP/PS2 mice. 5-CQA also dose-dependently upregulated *LRP1* mRNA in brain microvessel endothelial cells (data not shown). These findings also support the notion that the beneficial effects of 5-CQA on the brain are mediated, at least in part, by *LRP1* regulation. In the present study, we detected no difference in *LRP1* expression between the WT and APP/PS2 mice. Another study reported that LRP1 is locally upregulated in the astrocytes and neurons surrounding the Aβ plaques of AD patients [43]. Localized *LRP1* regulation might affect overall hippocampal *LRP1* expression in WT and APP/PS2 mice. Additionally, we were also unable to assess the effect of 5-CQA on LRP1 protein expression in hippocampus. Future research should evaluate detailed LRP1 expression analyses including protein expression of neuron, astrocytes, and vascular endothelial cells.

Accumulating evidence suggests that the paravascular pathway, which is also referred to as glymphatic system, facilitates cerebrospinal fluid (CSF) circulation through the brain parenchyma, promotes interstitial fluid (ISF)-CSF exchange, and enhances the clearance of interstitial solutes including Aβ [44,45]. Impairment of glymphatic fluid transport and Aβ clearance with advancing age were reported for APP/PS1 transgenic AD model mice [46]. Therefore, the glymphatic system may be an important alternate route for Aβ elimination in the brain besides BBB-mediated clearance. AQP4 water channels are polarized to perivascular astroglial endfeet and generate bulk ISF flow for Aβ clearance [45]. Reduced perivascular CSF-ISF exchange and interstitial Aβ clearance are related to decreased perivascular AQP4 polarization in elderly rodents [47]. AQP4 deletion in a mouse AD model exacerbated Aβ deposition and cognitive impairment [33]. Impaired perivascular AQP4 polarization was observed in AD patients and was strongly associated with AD status [48]. These findings suggest that AQP4 mislocalization reduces Aβ clearance and promotes Aβ plaque formation. Here, we showed that perivascular AQP4 polarization was significantly decreased in APP/PS2 mice but chronic 5-CQA administration restored AQP4 polarization around the vessels. These results suggest that 5-CQA facilitates the glymphatic system by maintaining perivascular AQP4 polarization which, in turn, reduces Aβ deposition in the brain. In the present study, we demonstrated that 5-CQA upregulated *LRP1* and controlled perivascular AQP4 polarization. There are few reports on the relationship between LRP1 and AQP4. Astrocytic LRP1 expression and Aβ transport function were inhibited in *AQP4*-null mice [49]. LRP1 expression was also reduced in the astrocytes of *AQP4*^−/−^APP/PS1 mice [33]. Therefore, AQP4 could affect LRP1 expression, 5-CQA might influence AQP4 localization, and both AQP4 and 5-CQA may upregulate *LRP1*. However, little is known about the underlying mechanisms regulating AQP4 polarization. Moreover, we were unable to measure brain Aβ clearance mediated by LRP1 or the glymphatic system. Further research is warranted to elucidate the mechanisms by which 5-CQA regulates AQP4 polarization, induces *LRP1*, and decreases Aβ plaques in the brain.

APP/PS2 mice presented with Aβ accumulation at age 2–3 mo [32] and cognitive dysfunction after age 4–5 mo [50]. In humans, Aβ accumulation has been observed 15–20 y before the onset of AD [51]. The failure of a recent clinical trial suggested that early intervention is important in the prevention of AD onset. In the present study, 5-CQA was administrated at 10 wk when Aβ deposition began but before the manifestation of cognitive dysfunction. Thus, 5-CQA helps prevent or delay AD onset. In our previous study, we indicated that 5-CQA in CPP computationally bound Aβ protofilaments and disaggregated Aβ fibrils in vitro [30]. The combined action of Aβ disaggregation and clearance by 5-CQA might be effective to treat a more severe phenotype after the onset of cognitive dysfunction.

Our preliminary experiments showed that feruloylquinic acids (3-FQA, 4-FQA, and 5-FQA) did not disaggregate Aβ fibrils in vitro (data not shown). Furthermore, diCQAs (3,4-diCQA, 3,5-diCQA, and 4,5-diCQA) were not detected in the plasma after coffee ingestion [20]. These findings support the concept that the beneficial effect of 5-CQA in CPP consists mainly of neuronal protection. It was reported that CGAs have antioxidant activity [27], promote neurite growth [22], and confer protection against Aβ-induced toxicity [23,24]. These salutary effects of CGAs may have also contributed to the neuronal protection and cognitive improvement observed in the present study. In this study, 5-CQA reduces Aβ accumulation in the brain by modulating molecules regulating Aβ clearance such as *LRP1* and AQP4. Recent evidence has shown that impaired Aβ clearance is a major characteristic of late-onset AD. For this reason, the enhancement of Aβ clearance is an efficacious therapeutic strategy for AD. Therefore, 5-CQA is an attractive agent for the prevention of cognitive dysfunction. Various dietary sources of polyphenols such as green tea catechin, curcumin, and blueberry have shown beneficial effects on cognitive function in animals and humans [52,53,54]. Here, we were unable to perform experiments to compare 5-CQA with the aforementioned polyphenols and/or test them in combination. Further investigations are required to evaluate the efficacy of 5-CQA. CGAs have been consumed by humans for a long time and have improved cognitive function in the elderly [29]. The pharmacological potential of 5-CQA in humans must also be assessed under clinical trial conditions.

## 5. Conclusions

Long-term 5-CQA administration enhanced *LRP1* expression, normalized AQP4 perivascular localization, reduced Aβ deposition in the brain, and ameliorated cognitive dysfunction in APP/PS2 mice. These findings suggest that CGAs may have beneficial effects on cognitive dysfunction in AD.

## Figures and Tables

**Table 1:** Effects of 5-CQA on body weight and food intake.

Parameter	WT	APP/PS2	APP/PS2 + 5-CQA
Body weight (g)	40.47 ± 0.97	38.55 ± 0.85	36.79 ± 0.65 ^##^
Food intake (kcal)	168.0 ± 3.2 ***	194.5 ± 3.5	188.4 ± 3.2 ^###^

Data are means ± SEM (*n* = 23–25 mice/treatment). ***, *p* < 0.001 vs. APP/PS2, ^##^, *p* < 0.01; ^###^, *p* < 0.001 vs. WT (Bonferroni’s post-hoc test).

**Table 2:** Effects of 5-CQA on mRNA expression in the mouse brain.

Gene Name and Brain Region		WT	APP/PS2	APP/PS2 + 5-CQA
Amyloid processing				
*ADAM10*	Cortex	0.92 ± 0.04	1.00 ± 0.05	0.93 ± 0.04
	Hippocampus	0.89 ± 0.02	1.00 ± 0.04	0.95 ± 0.04
*APP* (human)	Cortex	N.T.	1.00 ± 0.09	1.20 ± 0.06
	Hippocampus	N.T.	1.00 ± 0.09	1.18 ± 0.07
*BACE1*	Cortex	0.91 ± 0.04	1.00 ± 0.03	0.94 ± 0.04
	Hippocampus	0.87 ± 0.03	1.00 ± 0.04	1.08 ± 0.05^##^
Amyloid degrading				
*IDE*	Cortex	0.97 ± 0.04	1.00 ± 0.03	1.02 ± 0.05
	Hippocampus	1.01 ± 0.04	1.00 ± 0.03	1.01 ± 0.02
*NEP*	Cortex	1.06 ± 0.17	1.00 ± 0.13	1.17± 0.20
	Hippocampus	0.91 ± 0.05	1.00 ± 0.05	1.02 ± 0.05
Amyloid transport protein				
*LRP-1*	Cortex	0.93 ± 0.04	1.00 ± 0.04	1.03 ± 0.02
	Hippocampus	0.92 ± 0.03	1.00 ± 0.04	1.23 ± 0.09*, ^##^
*RAGE*	Cortex	1.43 ± 0.11*	1.00 ± 0.10	1.16 ± 0.09
	Hippocampus	1.19 ± 0.08	1.00 ± 0.10	1.27 ± 0.09

Values represent the relative gene expression levels for seven mice (means ±SEM) (APP/PS2 = 1). Differences were considered significant at *p* < 0.05. *: *p* < 0.05 vs. APP/PS2, #: *p* < 0.05, ##: *p* <0.01 vs. WT (Bonferroni’s post-hoc test). N.T., not tested; *ADAM10*, a disintegrin and metallopeptidase domain 10; *BACE1*, beta-site APP cleaving enzyme 1; *IDE*, insulin-degrading enzyme; *NEP*, neprilysin; *LRP-1*, low-density lipoprotein receptor-related protein 1; *RAGE*, receptor for advanced glycation end products.

## Supplementary Materials

The following are available online at https://www.mdpi.com/2072-6643/12/2/494/s1, Table S1: Taqman probes.
